# P333: Understanding patient safety in Angola: a situational analysis at hospital Américo Boavida

**DOI:** 10.1186/2047-2994-2-S1-P333

**Published:** 2013-06-20

**Authors:** JC Correia, L Antunes, JD Hightower, SB Syed

**Affiliations:** 1 Health, Permanent Mission of Angola, Geneva, Switzerland; 2 Americo Boavida Hospital, Luanda, Angola; 3 African Partnerships for Patient Safety , World Health Organization, Harare, Zimbabwe; 4 African Partnerships for Patient Safety, World Health Organization, Geneva, Switzerland

## Introduction

There is an increasing commitment in the African Region towards patient safety (PS) in hospitals, following acknowledgement that it is an important public health issue which needs to be addressed in order to improve population health.

## Objectives

To define the state of PS at Américo Boavida Hospital (HAB) in Luanda and thus enhance understanding of PS in Angola.

## Methods

Patient Safety Situational Analysis tool co-developed by WHO African Partnerships for Patient Safety was utilized. Data was gathered by a hospital team based on responsibilities in each of 12 PS areas, coordinated by an analysis lead. Data were analysed in each PS area, with a specific focus on infection prevention & control (IPC).

## Results

Approximately two-thirds of PS questions asked were answered affirmatively for HAB. For IPC approximately 60% of questions had a positive response .Specific findings include: 1. multidisciplinary IPC hospital team but no doctor or nurse assigned full time to activities; 2. occupational medicine service conducts health worker IPC training; 3. adequate reliable supply of soap and alcohol based handrub; 4. hospital posters on hand hygiene and IPC; 5. microbiology service with capacity for microscopy and aerobic culture; 6. lack of triaging and isolation policy of patients at high risk of transmission of infections.

In other areas, PS knowledge & learning, as well as PS & systems development had 90% positive responses. In medication safety, health worker protection and PS research & surveillance, affirmative responses averaged 80%. Health care waste management, linkage with national policies, PS funding and partnerships for PS revealed affirmative answers to questions posed of 60%, 50%, 40% and 25% respectively.

## Conclusion

Self-reported data quality is subject to bias. However, self-assessment can catalyze internally driven hospital improvement. This first analysis serves as a robust PS baseline to plan hospital action to address shortcomings. Repeat PS situational analysis can track progress. The approach can also be replicated across hospitals in Angola to guide national action. Further, the areas of strength at HAB (identified through the PS analysis) can serve as a resource for hospitals in Angola.

## Disclosure of interest

None declared

